# Expression of Fbxo7 in Haematopoietic Progenitor Cells Cooperates with p53 Loss to Promote Lymphomagenesis

**DOI:** 10.1371/journal.pone.0021165

**Published:** 2011-06-17

**Authors:** Mikhail Lomonosov, El Kahina Meziane, Hongtao Ye, David E. Nelson, Suzanne J. Randle, Heike Laman

**Affiliations:** Department of Pathology, University of Cambridge, Cambridge, United Kingdom; University of Sao Paulo – USP, Brazil

## Abstract

Fbxo7 is an unusual F box protein that augments D-type cyclin complex formation with Cdk6, but not Cdk4 or Cdk2, and its over-expression has been demonstrated to transform immortalised fibroblasts in a Cdk6-dependent manner. Here we present new evidence *in vitro* and *in vivo* on the oncogenic potential of this regulatory protein in primary haematopoietic stem and progenitor cells (HSPCs). Increasing Fbxo7 expression in HSPCs suppressed their colony forming ability *in vitro*, specifically decreasing CD11b (Mac1) expression, and these effects were dependent on an intact p53 pathway. Furthermore, increased Fbxo7 levels enhanced the proliferative capacity of p53 null HSPCs when they were grown in reduced concentrations of stem cell factor. Finally, irradiated mice reconstituted with p53 null, but not wild-type, HSPCs expressing Fbxo7 showed a statistically significant increase in the incidence of T cell lymphoma *in vivo*. These data argue that Fbxo7 negatively regulates the proliferation and differentiation of HSPCs in a p53-dependent manner, and that in the absence of p53, Fbxo7 expression can promote T cell lymphomagenesis.

## Introduction

Fbxo7 is a member of a protein family defined by the presence of an F-box domain, which binds the Skp1 protein [1]–[3]. Through this interaction, F-box proteins are recruited as components of SCF-type E3 ubiquitin ligases, which target proteins for ubiquitination [4]–[8]. Fbxo7 has been reported to catalyse the ubiquitination of HURP/DLG7, a regulator of mitotic spindle assembly, marking the protein for degradation by the proteasomes [9]. Fbxo7 also promotes ubiquitination of cIAP1, an inhibitor of apoptosis family member which regulates canonical and non-canonical NF-κB signalling [10]. Fbxo7 also has been shown to interact directly with the cell cycle regulators, p27 and Cdk6, and stabilize their association with D-type cyclins [11]. Both cyclin D and Cdk6 are proto-oncogenes, and as Fbxo7 increases this kinase activity, it is also a putative proto-oncogene. In support of this idea, the over-expression of Fbxo7 in immortalised fibroblasts augmented the levels of Cdk6 bound to D-type cyclins and led to transformation [11], [12]. These cells were invasive, capable of anchorage-independent growth, and formed tumours when injected subcutaneously into athymic nude mice. Moreover, Fbxo7-mediated transformation of NIH3T3 cells was Cdk6-dependent, as siRNA-mediated reduction of Cdk6 reversed transformed phenotypes [11].

This ability of Fbxo7 to transform immortalised fibroblasts raised questions regarding the potency, cellular context, and mechanisms by which Fbxo7 may act as an oncogene. Cdk6 is the predominant G1 kinase in haematopoietic cells [13], where it plays an important role in proliferation and in negatively regulating terminal differentiation [14]–[18]. In support of this, mice lacking Cdk6 have thymic and splenic hypoplasia and mild defects in haematopoiesis [19], [20]. Conversely, the amplification of Cdk6 is associated with splenic marginal zone lymphoma and B cell chronic lymphocytic leukaemia [21], [22]. Because our previous data indicated a direct and specific relationship between Fbxo7 and Cdk6, we assayed the functional effects of Fbxo7 expression in foetal liver (FL)-derived, primary haematopoietic stem and progenitor cells (HSPC). Fbxo7 was retrovirally transduced into wild-type (WT) and p53 null cells, and its effects *in vitro* on lifespan, colony formation, cell proliferation and differentiation, as well as its ability to promote tumourigenesis *in vivo* were assayed. Our results demonstrate that increased Fbxo7 expression suppressed colony formation capacity and altered the differentiation of HSPCs, while stimulating proliferation and lymphomagenesis. These data argue that Fbxo7 has oncogenic activity and that this is largely dependent on the growth conditions and p53 status of the cell.

## Results and Discussion

### Fbxo7 decreased colony formation by HSPCs, in a p53-dependent manner

**Figure 1:**
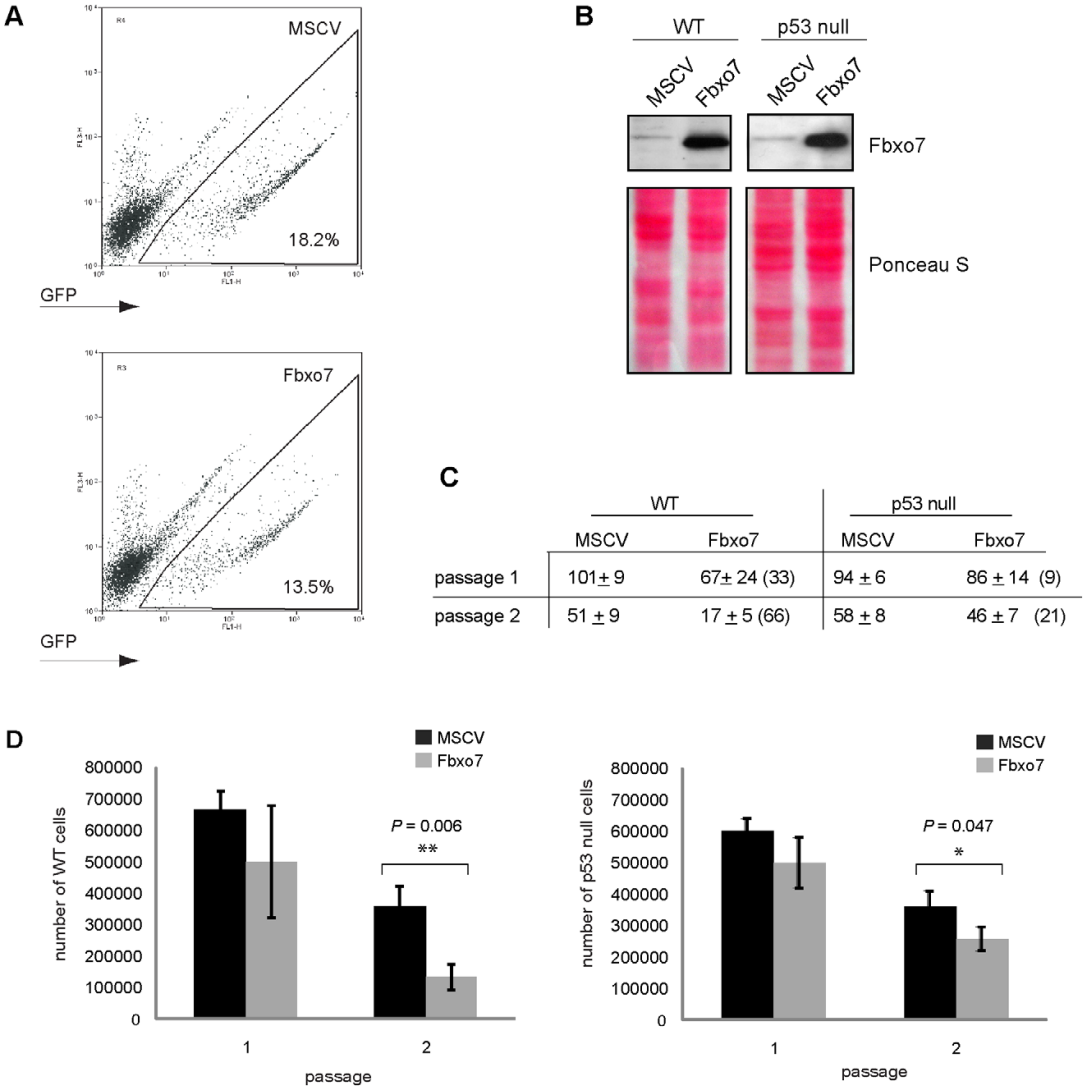
Fbxo7 expression reduced the colony forming capacity and number of WT and p53 null cells. (**A**) Representative FACS plots of retrovirally infected FL cells showing GFP expression. (**B**) Expression of Fbxo7 in cells assayed by immunoblotting and Ponceau S staining as a loading control. (**C**) Table of quantification of the number of colonies at each passage. Number in parentheses is the percent decrease relative to the MSCV control. Error is represented as the SD. Quantification is of two independent experiments for each cell type. (**D**) Graphs of the quantification of total numbers of either WT (left) or p53 null (right) cells at each passage. Error is represented as the SD. Quantification is of two independent experiments for each cell type. * denotes statistical significance, *P* value<0.05; ** *P* value<0.01.

As a source of HSPCs, FLs were harvested from E13.5 mouse embryos and infected with recombinant retroviruses expressing either GFP or human Fbxo7-IRES-GFP from the MSCV promoter. GFP^+^ cells were collected by flow cytometry (Figure 1A), and immunoblotting of these cell lysates demonstrated the expression of endogenous Fbxo7 in both WT and p53 null HSPCs and also the robust expression of the transduced human Fbxo7 (Figure 1B). The effect of Fbxo7 expression in HSPCs was tested by *in vitro* colony formation assays. Equal numbers of GFP^+^ cells were seeded into media promoting growth and differentiation along the granulocyte/macrophage (G/M) lineage. After 10–14 days, both the total number of cells per well and the number of colonies per well were counted as measures of proliferative capacity and colony forming capacity, respectively. The effect of Fbxo7 expression was compared to the MSCV control in both WT and p53 null cells using a serial replating assay. Despite some variability, there was a consistent reduction in the colony forming capacity of Fbxo7 expressing cells as compared to the MSCV control, and a commensurate decrease in the total number of cells per well. On the first plating, Fbxo7 expression caused a 33% reduction on average in the number of colonies formed by WT cells (Figure 1C) and a 25% reduction in the total number of cells (Figure 1D). In p53 null cells, however, Fbxo7 expression caused only an average 9% reduction in colony number and 17% reduction in total number of cells. On the second replating, the expression of Fbxo7 in WT cells reduced the number of colonies and the total number of cells by 66% and 63%, respectively. In p53 null cells, Fbxo7 expression reduced colony number by 21% and total cell number by 29% on the second replating. The *P* values for the effect of Fbxo7 expression on the total number of cells per well on the second replating were significant at 0.006 for WT cells and 0.047 for p53 null cells (Figure 1D). We also observed that neither WT nor p53 null cells expressing MSCV or Fbxo7 were capable of being replated more than 3 times indicating that the lifespan of these *in vitro* cultured HSPCs had not been altered in these experiments. These data demonstrate that the expression of Fbxo7 had a suppressive effect on colony forming capacity of WT HSPCs and to a lesser extent, p53 null cells. This suggests that in WT cells, Fbxo7 expression activated a p53-dependent response, which limited colony formation.

**Figure 2:**
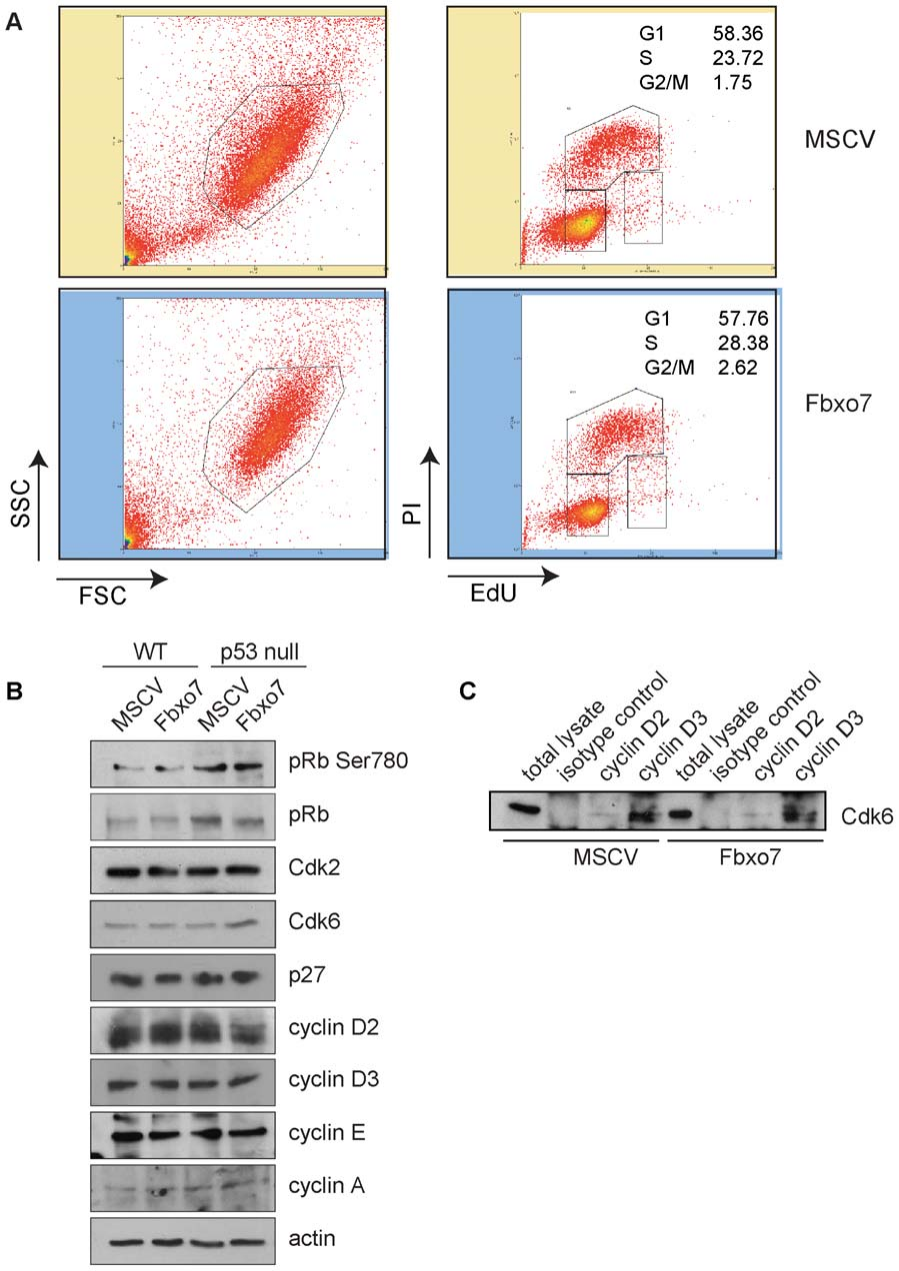
Fbxo7 expression enhances the proliferation of p53 null cells grown at a reduced SCF concentration. (**A**) FACS plot of EdU incorporation, showing forward scatter (FSC) on the x-axis and (SSC) side scatter on the y-axis for the left hand panels, with the gated population used for EdU analysis and anti-EdU conjugated to Alexafluor647 on the x-axis and propidium iodide intensity on the y-axis for the right hand panels. (**B**) Immunoblotting for the expression of various cell cycle regulators, as indicated, in WT and p53 null cells expressing Fbxo7 or the empty MSCV vector. (**C**) Immunoblotting for Cdk6 in immunoprecipitations of D-type cyclins from equal numbers of sorted WT cells expressing either Fbxo7 or the empty MSCV vector.

It was possible that the suppression of colony formation caused by Fbxo7 expression of might be due to an effect on the cell cycle. The stronger effect was seen in WT HSPCs, so GFP^+^ WT HSPCs expressing either the MSCV control or Fbxo7 retroviral vector were sorted and seeded as above. Five days later, cells were pulse-labelled with EdU, harvested and stained with propidium iodide to enable the identification of G1, S or G2/M phase populations by FACS analysis. No significant differences in the percentages of cells in each phase were observed between MSCV and Fbxo7 expressing cells (Figure 2A), indicating that Fbxo7 had not altered the cell cycle of HSPCs. To investigate the possible effects of Fbxo7 expression on cell cycle regulators, protein lysates were produced from sorted control and Fbxo7-expressing WT and p53 null cells, which were assayed for the effects on the levels of G1 and S phase cell cycle regulators. No significant changes were observed in the total levels of D-type cyclins, cyclins E and A, Cdk2 and Cdk6 or p27 (Figure 2B). To assess whether the expression of Fbxo7 in sorted HSPCs altered the levels of Cdk6 associated with D type cyclins, lysates made from equal numbers of retrovirally infected GFP^+^ WT HSPCs were immunoprecipitated with antibodies to cyclins D2 and D3, and immunoblotted for the presence of Cdk6. However, the amount of Cdk6 co-immunoprecipitating with D-type cyclins was unchanged (Figure 2C). In addition, the levels of phosphorylation on serine 780, a D-cyclin/Cdk specific site, in the retinoblastoma protein were unchanged (Figure 2B). These data indicate that the levels of cyclin D/Cdk6 complexes, activity, and the entry into S phase were not affected by Fbxo7 expression in HSPCs.

### Fbxo7 affected G/M, but not erythroid, differentiation

**Figure 3:**
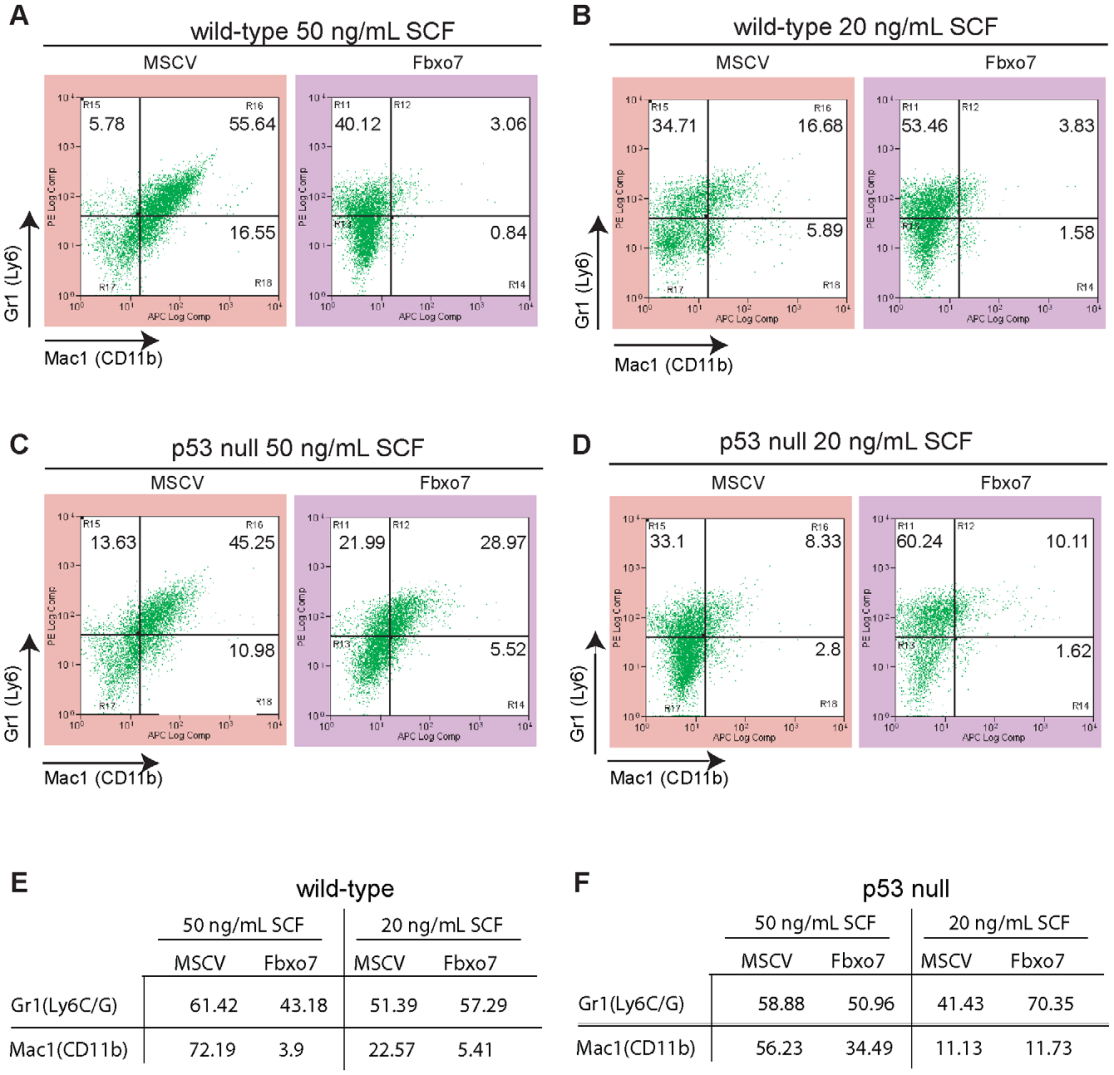
Fbxo7 expression alters the expression of markers of myeloid differentiation. FACS analysis of immunostaining for Mac1 (CD11b) on the x-axis and Gr1(Ly6C/G) on the y-axis for WT cells grown at 50 ng/mL (**A**) or 20 ng/mL (**B**) of SCF. FACS analysis of immunostaining for Mac1 (CD11b) and Gr1(Ly6C/G) for p53 null cells grown at 50 ng/mL (**C**) or 20 ng/mL (**D**) of SCF. Table of percentages of either Mac1 (CD11b) or Gr1(Ly6C/G) positive cells for WT cells (**E**) and p53 null cells **(F**).

Fbxo7 expression had a strong suppressive effect on colony formation and proliferation of HSPCs, so we next wanted to determine whether its expression also affected their differentiation. Equal numbers of retrovirally infected FL-derived HSPCs were seeded and incubated under standard growth conditions. After 14 days, cells were harvested and immunostained for the expression of cell surface markers Ly6C/G (Gr1), which is a myeloid differentiation antigen whose expression increases during granulocyte differentiation, and CD11b (Mac-1), which is a macrophage differentiation factor. We observed that 55.64% of WT cells expressing the MSCV control were Ly6C/G^+^ CD11b^+^ (Figure 3A). As SCF levels can affect the differentiation of G/M progenitor cells and enhance their response to other cytokines [23], we also tested the effect of reducing the SCF concentration to 20 ng/mL, and this decreased the Ly6C/G^+^ CD11b^+^ population to 16.68% (Figure 3B). Fbxo7 expression in WT HSPCs strongly inhibited the appearance of the double positive population, which accounted for only 3.06% and 3.83% of the cells, at 50 and 20 ng/mL of SCF, respectively. We noted that the effect of Fbxo7 expression was attributable mainly to a decrease in CD11b expression as Ly6C/G was still expressed and even slightly enhanced when Fbxo7 was introduced (Figure 3A, B, E).

When identical experiments were performed using MSCV transduced p53 null cells, the number of double positive cells was reduced by 19% compared to WT cells (comparing left-most panels in 3A and 3C), even though p53 null cells formed colonies and proliferated as well as WT HSPCs when grown in 50 ng/mL SCF (Figure 1C, D). The expression of Fbxo7 in p53 null cells also reduced the double positive Ly6C/G^+^ CD11b^+^ population to 29% compared to the 45% seen in the MSCV control (Figure 3C). However, when measured against the strong suppressive effect that Fbxo7 expression had on the Ly6C/G^+^ CD11b^+^ population in WT HSPCs, this amounted to a 9.5 fold increase in the double positive population in p53 null cells expressing Fbxo7 (comparing right-most panels in 3A and 3C). This suggests that the Fbxo7 suppression of CD11b expression was p53 dependent. As with WT cells, when p53 null cells were grown in 20 ng/mL SCF, the double positive population was sharply reduced to 8.33%, and this was also due mainly to a lack of CD11b expression (Figure 3D). These data imply that SCF promoted CD11b expression, enabling myeloid differentiation. Finally, the expression of Fbxo7 in p53 null cells grown in 20 ng/mL SCF did not alter the double positive population significantly; however, the number of Ly6C/G^+^ cells increased from 41.43% to 70.35%. This contrast the effect of Fbxo7 expression when cells were grown in 50 ng/mL SCF which suppressed slightly the number of Ly6C/G^+^ cells from 58.88% to 50.96% (Figure 3F). This demonstrated that the effect of Fbxo7 on granulocytic differentiation was influenced strongly by the levels of SCF. These data showed that at higher SCF levels and p53 expression, Fbxo7 acted to suppress CD11b, whilst at lower SCF conditions in cells lacking p53, Fbxo7 strongly enhanced Ly6C/G^+^ expression. Therefore, these data indicated that the effect of Fbxo7 in HSPCs on G/M differentiation was determined both by the p53 status and the growth conditions in the assays.

We next tested the ability of Fbxo7 to affect differentiation along the erythroid lineage. WT and p53 null cells were seeded in standard conditions for erythropoiesis. No statistically significant differences were observed in the number of erythroid colonies formed by Fbxo7 expressing or by control HSPCs on either background, nor in the number of cells co-staining with anti-CD71 antibody, which recognizes transferrin receptor and Ter-119, which recognizes an erythroid specific marker, and which together delineate the later stages of erythroid lineages (negative data not shown). This suggests that the effects of Fbxo7 expression on colony formation, proliferation and differentiation *in vitro* were specific to HSPCs differentiated along the G/M lineage.

In sum these *in vitro* experiments showed that Fbxo7 affected the proliferation and differentiation of HSPCs along particular cell lineages, and moreover, its function was sensitive to specific growth and genetic conditions.

### Fbxo7 increased proliferation of p53 null HSPCs when stem cell factor (SCF) was reduced

**Figure 4:**
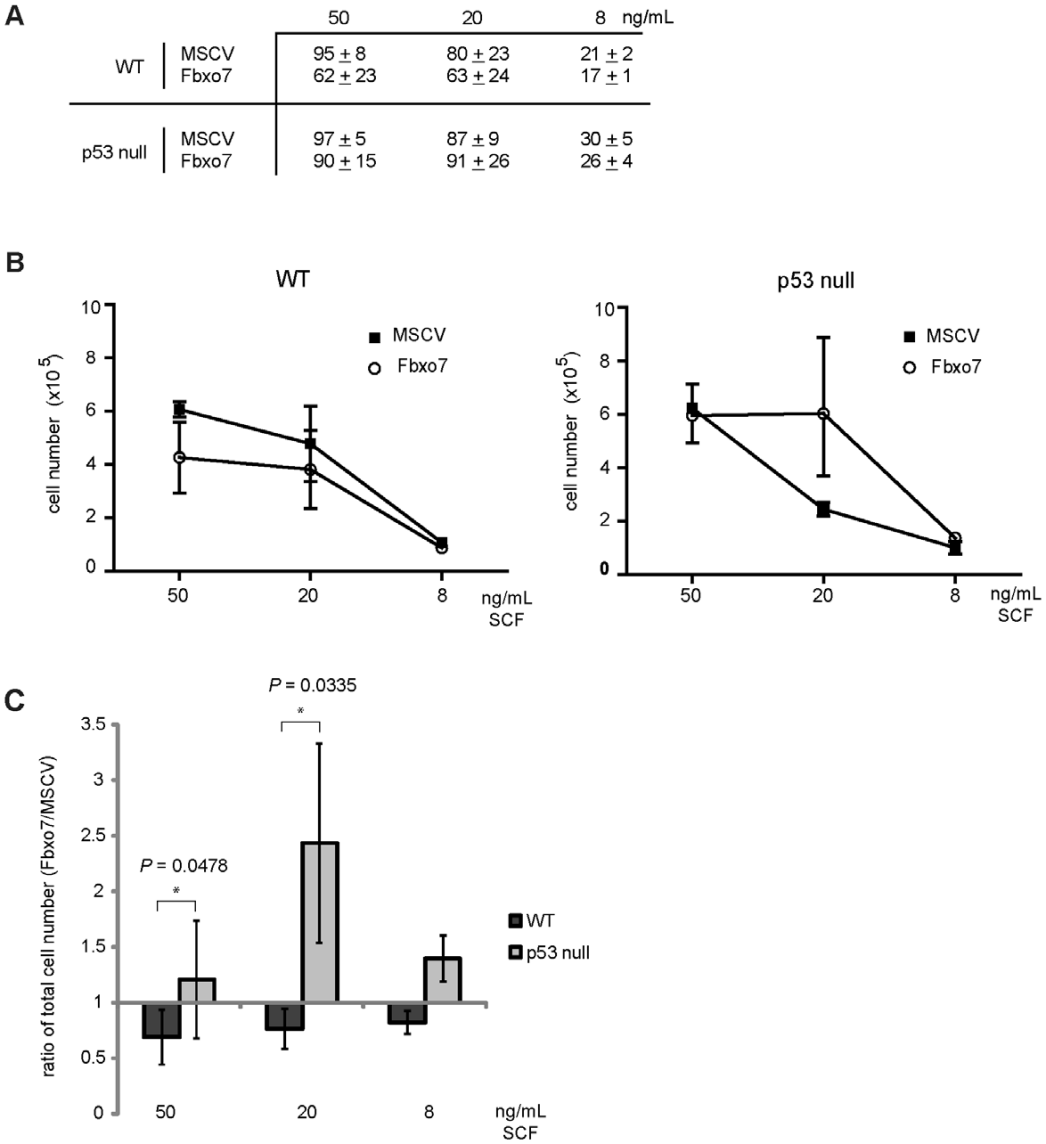
Fbxo7 acts as a proliferative factor in p53 nulls grown in reduced SCF. (**A**) Table of the quantification of colony number of WT and p53 null cells grown at different concentrations of SCF. (**B**) Graphs of the total cell number at three concentrations of SCF in WT and p53 null cells expressing Fbxo7 compared to MSCV. (**C**) Graph of ratio of the number of either WT or p53 null cells expressing Fbxo7 compared to MSCV at different concentrations of SCF. In these experiments, the error is represented as the SD, and quantification is of three independent experiments.

As SCF can act as a survival factor for progenitor cells with only limited effects as a mitogen [23], we next tested whether increasing Fbxo7 expression could compensate for reduced SCF signalling. In these experiments, the concentration of SCF was reduced, while FCS, IL-3 and IL-6 concentrations were maintained. As expected, the number and size of colonies formed by WT HSPCs was reduced when the SCF was decreased from 50 ng/mL (standard conditions) to 20 or 8 ng/mL (Figure 4A), and consistent with our results in Figure 1C, Fbxo7 reduced the number of WT HSPCs colonies under standard growth conditions. However, at lower SCF concentrations, Fbxo7 expression did not significantly alter the number of colonies formed by WT HSPCs (Figure 4A) or the total number of WT cells that grew (Figure 4B, C). We conclude that Fbxo7 exerts its inhibitory effect on colony formation at a threshold concentration of SCF above 20 ng/mL and that increased Fbxo7 expression did not substitute for reduced SCF signalling.

As with WT HSPCs, the number of colonies formed by p53 null HSPCs was also reduced when the concentration of SCF was reduced (Figure 4A). Moreover, Fbxo7 expression in p53 null HSPCs did not affect colony formation, irrespective of the SCF concentration. However, Fbxo7 expression increased the proliferation of p53 null cells when grown in 20 ng/mL SCF (Figure 4B). A comparison of the ratios of total cell numbers expressing Fbxo7 normalised to MSCV of either WT or p53 null cells showed that Fbxo7 expression provided a significant growth advantage to p53 null cells compared to WT (Figure 4C). Strikingly, the number of cells per colony, rather than colony formation, was specifically affected. Thus the average number of MSCV control cells per colony was approximately 2,800 as compared to 6,600 for Fbxo7-expressing cells, representing a 2.4 fold increase. These data demonstrate that Fbxo7 was capable of enhancing cell proliferation under specific genetic and growth conditions. In addition, these were the same genetic and cytokine concentrations in which Fbxo7 expression affected G/M differentiation to increase the proportion of Ly6C/G^+^ cells (Figure 3D). One further implication from these data is that Fbxo7 expression in p53 null HSPCs might be capable of substituting for cytokine signalling to relieve growth factor dependence.

### Fbxo7 contributed to tumour formation *in vivo*

**Figure 5:**
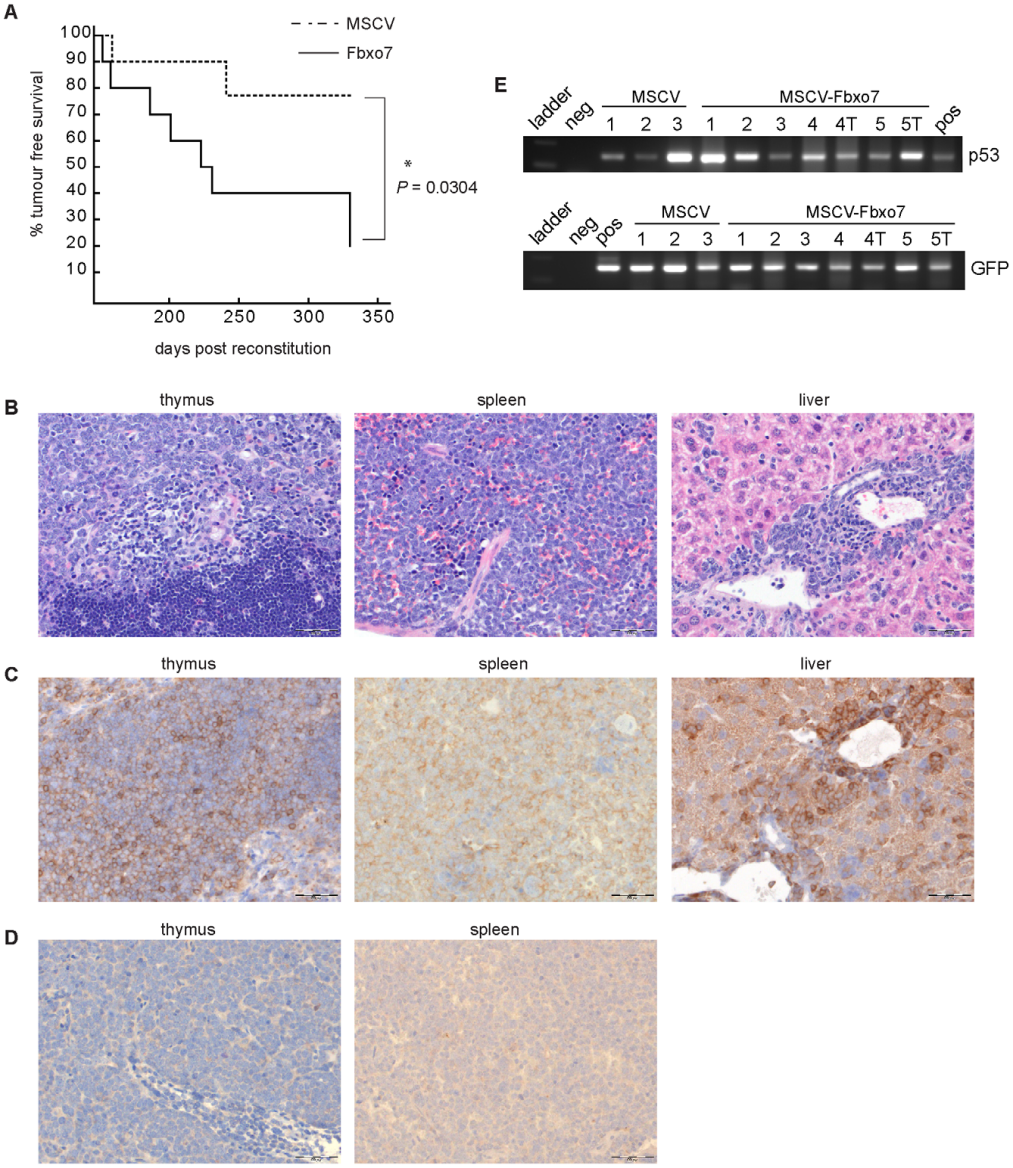
Fbxo7 cooperates with p53 mutation to promote lymphomagenesis *in vivo*. (**A**) Graph of Kaplan-Meier survival curve of mice reconstituted with p53 null FL cells infected with retroviruses expressing either MSCV control (n = 10, dashed line) or Fbxo7 (n = 10, solid line). (**B**) H&E staining and immunohistochemistry was conducted as previously described [11] for (**C**) CD3 and (**D**) Fbxo7 in tissue samples from mice reconstituted with Fbxo7-expressing cells. Size bar is 50 µm. (**E**) PCR amplification reactions for either the p53 null allele (top) or the GFP gene in the MSCV vector (bottom) performed on genomic DNA isolated from paraffin-embedded tissue samples from mice reconstituted with p53 null HSPCs infected with retroviruses bearing either the empty MSCV vector or the MSCV vector expressing Fbxo7. Numbered samples consist of pooled biopsies from multiple organs (liver, spleen, thymus, intestine, kidney, heart) from mice in the two different cohorts, as indicated. ‘T’ denotes samples which consisted of tumour tissue only. For positive controls for the PCR reactions, in the p53 reaction (top), ‘pos’ denotes a reaction where genomic DNA from a p53 null mouse was added, and for the GFP reaction (bottom) ‘pos’ denotes a reaction where MSCV plasmid DNA was added. ‘Neg’ denotes reactions where no template DNA was added.

We next tested whether increased Fbxo7 expression in HSPCs would promote tumourigenesis. WT and p53 null FL cells expressing either Fbxo7-IRES-GFP or the empty MSCV control vector were used to reconstitute irradiated mice. Prior to injection, the percentage of GFP^+^ cells was assayed and ranged from 2–15% for the Fbxo7-IRES-GFP vector infected populations and 4–20% for the vector control (Figure 1A). The mixed population of infected and uninfected cells was injected into irradiated mice. At 10 months post-reconstitution, all of the animals reconstituted with WT HSPCs expressing either MSCV or Fbxo7 were healthy (n = 10 each; negative data not shown). However, a significant number of animals reconstituted with p53 null cells showed signs of illness between 5 and 7 months post-reconstitution. These included 2 out of 10 mice reconstituted with cells expressing MSCV only and 7 out of 10 mice reconstituted with cells expressing Fbxo7. The two survival curves differed significantly, *P* = 0.0304. This difference in survival between the animals reconstituted with p53 null HSPCs expressing either MSCV or Fbxo7 indicated that Fbxo7 expression had a statistically significant influence on tumour-free survival time (Figure 5A). The two mice reconstituted with p53 null HSPCs expressing MSCV both had splenomegaly and one animal also had enlarged inguinal lymph nodes and tumour cells infiltrating the kidney. The mice reconstituted with p53 null HSPCs expressing Fbxo7 had splenomegaly and enlarged lymph nodes, and in addition, one animal had thymoma. H&E staining showed the presence of tumour cells in these immune tissues and also disseminated into other organs, including liver, lung, and kidney (Figure 5B). Immunohistochemical analysis demonstrated that tumour cells in both the MSCV and Fbxo7 samples were CD3^+^ CD20^−^, indicating a T cell lineage (Figure 5C and data not shown).

To assess whether the transplanted HSPCs had repopulated the immune systems of the mice and contributed to tumour formation, lymph nodes and spleens were analysed for GFP expression. Surprisingly, although spleens harvested from MSCV control animals showed the presence of GFP^+^ cells, ranging from 0.02% to 7%, no GFP^+^ cells were detected in spleen or tumour samples isolated from mice reconstituted with Fbxo7-p53 null HSPCs (negative data not shown). This was despite the fact that GFP expression had been established prior to injection (Figure 1A), and robust Fbxo7 expression was confirmed in HSPCs in parallel *in vitro* experiments (Figure 1B). Moreover, immunohistochemistry for Fbxo7 was performed on tissue sections containing tumour cells, and Fbxo7 expression was found to be either negative or only weakly positive (Figure 5D). These data raised the possibility that p53 null cells infected with the Fbxo7-IRES-GFP retroviruses either did not participate in long-term reconstitution or tumour formation, or alternatively, that there was a preferential down-regulation of transgene expression from the Fbxo7-IRES-GFP vector. To distinguish between these two possibilities, we tested directly whether DNA sequences from retrovirally-infected p53 null HSPCs could be detected in tissue and tumour samples by PCR analysis. Sequences from both the p53 null allele and from the GFP gene from the MSCV vector were detected (Figure 5E), confirming the presence of retrovirally transduced p53 null HSPCs. This therefore suggested that the lack of GFP expression in Fbxo7-derived tumour cells and the weak expression of Fbxo7 expression in tissue samples was due to the Fbxo7-IRES-GFP transgene being either only weakly expressed or silenced altogether. There is considerable evidence that the MSCV vector, although efficiently expressed in HSCs is also eventually subject to silencing by DNA methylation. Silencing can be reversed by treatment with 5-azadeoxycytidine demonstrating that DNA methylation directly participates in the repression of the promoter, and in fact, cytosine methylation of two clusters of CpGs in the MSCV LTR have been shown to be important for its transcriptional silencing [24]–[28]. Another possibility for the lack of GFP and Fbxo7 expression is that Fbxo7 had a ‘hit-and-run’ effect on tumour promotion, so that even though it was expressed initially in the injected HSPCs, its expression was down-regulated in the tumours. Moreover, the fact that GFP expression was detected in the tumour cells derived from the MSCV cohort of animals suggests that the tumours from animals in the Fbxo7 cohort did not require, or perhaps could not tolerate, continued Fbxo7 expression for proliferation or survival. This differs from our previous findings of the high expression of Fbxo7 seen in lung and colon cancer biopsies and in the transformation of fibroblasts [11]. However, this difference may reflect the fact that in epithelial cells, Fbxo7 may have its proto-oncogenic effect via the up-regulation of the Cdk6 activity whereas in HSPCs, Fbxo7 does not appear to increase cyclin D/Cdk6 activity, and the mechanism by which Fbxo7 promotes tumour formation is different.

The data presented here demonstrate that Fbxo7 can cooperate with p53 deletion to promote tumour formation originating from a primary stem or progenitor cell *in vivo*; however, as the time to tumour onset was 5–7 months, this implied that other events contributed to tumour formation. The mechanism by which Fbxo7 expression promoted lymphomagenesis in p53 null HSPCs did not appear to be mediated by increasing Cdk6 activity or levels. Cdk6 has an important role in haematopoiesis being highly expressed in the stem and progenitor compartment, but then down-regulated in lineage committed cells [14]. For example, Cdk6 has a specific role in the differentiation of myeloid and erythroid lineages and must be down-regulated to allow terminal differentiation [14], [17]. One regulatory function for Cdk6 in the myeloid lineage is to prevent the Runx1 transcription factor from binding promoters which impose lineage-specific differentiation [14]. We speculate that abundant expression of Fbxo7 might inappropriately stabilize this non-canonical, kinase-independent function of Cdk6 as cells proceed through lineage specification. This extra Cdk6 activity might then promote expansion and/or proliferation of specific populations of maturing cells or prevent their terminal differentiation. As the tumours which arose were CD3 positive, a simple hypothesis is that this occurred in the T cell lineages *in vivo*. However, an alternate possibility is that this occurred in the myeloid lineage and tumours arose via an indirect mechanism. Our data also demonstrated that Fbxo7 had a robust and specific effect on myeloid differentiation in a p53 null setting grown at a standard SCF concentration. This combination of factors resulted in an increase in double positive (Ly6C/G^+^ CD11b^+^) cells. It is therefore possible that the ability of Fbxo7 to affect G/M differentiation modulated the appearance and lifespan of myeloid-derived suppressor cells (MDSC) to promote tumour formation *in vivo*. MDSCs are a heterogeneous population of immature myeloid cells, which suppress T cell functions, inhibiting both the innate and adaptive immune responses [29], [30]. This dampened T cell response curbs the host's immune surveillance and allows tumour progression [29], [31], [32]. Normally, host- and/or tumour-derived cytokines from the tumour micro-environment dictate the numbers of MDSCs. However, in our experiments using HSPCs, one possibility is that increased Fbxo7 expression disrupted normal myelopoiesis and increased the numbers of immature myeloid cells in a cell intrinsic manner. Moreover, Fbxo7 modulation of NF-κB signalling through ubiquitination of cIAP1 [10] might provide an additional pro-inflammatory signal, which is also hypothesized to contribute to the genesis and activation of MDSCs [33]. Thus one indirect model for results obtained in the *in vivo* lymphomagenesis assays is that a low level of lymphomagenesis (20%) is seen upon reconstitution of irradiated animals using retrovirally-infected, p53 null HSPCs. These lymphomas may be due to insertional mutagenesis of retroviral sequences coupled with an inability to undergo apoptosis due to a lack of the p53 pathway. Reconstitution with Fbxo7-expressing HSPCs caused an increase in MDSCs levels which subsequently reduced host immune surveillance to increase the levels of lymphomagenesis (70%). One way to address this would be to determine whether the mice have MDSCs bearing the Fbxo7 transgene.

Another possible mechanism for Fbxo7-mediated lymphomagenesis is that other substrates of Fbxo7, reliant upon its ubiquitinating activity, contributed directly to tumour formation specifically in a p53 background. p53 has an established role in maintaining low levels of basal NF-κB activity, as evidenced by the fact that p53 null mice show hyperactive inflammatory and immune responses [34], [35]. As mentioned above, one ubiquitination substrates of Fbxo7 is cIAP1 [10], which is a regulator of NF-κB signaling [36]–[38]. cIAP1 has also been shown to inhibit non-canonical NF-κB activity by ubiquitinating NF-κB-inducing kinase (NIK), a kinase that activates inhibitor κB kinase (IKK) which subsequently triggers the processing of non-canonical NF-κB transcription factors to their active form. We speculate that Fbxo7 expression stimulated the degradation or inactivation of cIAP1, stabilizing NIK and triggering the constitutive activation of the non-canonical NF-κB transcription factors. In wild type HSPCs expressing Fbxo7, this activation of the NF-κB pathway alone was insufficient to induce lymphoma. However, in the absence of p53 and with the expression of Fbxo7 further increasing already elevated NF-κB signalling, these mutations cooperated to induce lymphoma in T cells. The non-canonical NF-κB pathway may be especially important to the oncogenesis of T cell lymphoma, as in human T cell lymphomas its activation is evident and has been shown to correlate with resistance to chemotherapy and poor survival [39]. Lastly we note that although the pivotal roles of p53 and NF-κB pathways in cancer are robustly documented, rather than having a simply synergistic or antagonistic relationship, the crosstalk between them is highly specific to both cellular context and external/internal stimuli [40]. We observed that the functional effects of Fbxo7 expression were highly cell type-specific and exquisitely sensitive to cytokine concentration, suggesting the involvement of these pathways.

In sum, we report the effects of Fbxo7 over-expression in primary, murine WT and p53 null HSPCs from FL under different growth conditions. Our studies suggest that the effect of Fbxo7 expression is highly context-dependent and is influenced by factors, like cell type, cytokine concentration and p53 status. In addition, Fbxo7 can promote tumour formation from stem and progenitor cells of this tissue. Our experiments argue that Fbxo7 has an important and sensitive role in balancing the proliferative and differentiation capacities, and it possesses proto-oncogenic activity in p53-deficient haematopoietic cells.

## Materials and Methods

### Construction and production of recombinant retroviruses

Retroviruses were constructed in the murine stem cell virus backbone which was generously provided by Scott Lowe [41]. Human Fbxo7 (isoform 1) cDNA was amplified by PCR and subcloned into MSCV-IRES-GFP. Clones were fully sequenced prior to use. Plasmid DNA constructs encoding either ‘empty’ MSCV-IRES-GFP or MSCV-Fbxo7-IRES-GFP retroviral vectors were co-transfected with an ecotropic packaging vector [42] into EcoPhoenix cells generously provided by Gary Nolan. At 48 h post-transfection, viral supernatants were filtered and used for infection of FL-derived HSPCs. Freshly isolated WT or p53 null foetal liver (FL) cells from E13.5 embryos were seeded into 6-well plates at a density of 2×10^6^ cells per well in 2.5 mL of media (40% DMEM, 40% IMDM, 4 mM L-Glutamine, 0.1 mM β-mercaptoethanol, 16% fetal bovine serum, 4% WEHI-3B conditioned media, 20 ng/mL SCF, 2 ng/mL IL-6, 0.2 ng/mL IL-3; all cytokines from Fitzgerald Laboratories). 24 hrs after seeding, cells were infected with retroviral supernatants supplemented with 4 µg/mL polybrene. Infections were repeated 3 times in 48 hrs. GFP^+^ cells were isolated by FACS using a Dako-Cytomation MoFlo sorter for the indicated experiments.

### Immunoprecipitation and Western analysis

Equal numbers of sorted GFP^+^ cells were lysed in HB buffer (50 mM Hepes, pH 7.4, 150 mM NaCl, 20 mM EDTA, 1 mM DTT, 10 mM β-glycerophosphate, 0.5% Triton-X-100, 10 mM NaF and protease inhibitor cocktail (Sigma Aldrich). Lysates were pre-cleared with Protein A/G Plus Agarose (Santa Cruz Biotechnology (SCBT)) and then incubated at 4°C for 4 hrs with normal mouse IgG (SCBT, sc-2025), anti-cyclin D2 (SCBT, sc-181) or anti-cyclin D3 antibodies (SCBT, sc-6283). Immunoprecipitated proteins were captured using Protein A/G Plus agarose beads and washed with HB buffer before being denatured in Laemmli buffer, resolved by SDS-PAGE and analyzed by western blotting for Cdk6 (SCBT, sc-177). Input proteins were also analysed for cell cycle regulatory proteins by western blotting, including pRb Ser780 (Cell Signaling #9307), pRb (Becton Dickinson (BD) Pharmingen, 554136), Cdk2 (BD Pharmingen, 15536E), p27 (SCBT, sc-528), cyclin E (SCBT, sc-481), cyclin A (SCBT, sc-751) and actin (Sigma Aldrich, A2066).

### Colony formation assays

Infected cells were collected by FACS sorting, counted on a CASY cell counter (Scharfe Systems), and 4,500 cells were seeded in triplicate in 1.1 mL of MethoCult methylcellulose-based media (Stem Cell Technologies) per 35 mm well, as per manufacturer's protocol. For G/M differentiation, media was supplemented with cytokines at the following “standard” concentrations: 50 ng/mL stem cell factor (SCF), 10 ng/mL IL-3, 10 ng/mL IL-6. Colony number and the total number of cells per well were counted 10 to 14 days later. Total numbers of cells were counted by extracting all cells in a well. This was performed by diluting colonies embedded in methylcellulose with 15 volumes of PBS, and centrifuging at 300 g for 8 mins at RT. Cell pellets were washed, and cell number determined on a CASY cell counter. For serial replating, 4,500 cells were reseeded in methylcellulose as above. For erythroid differentiation, media was supplemented with 2 units/mL of erythropoietin (Sigma Aldrich), and colonies were counted 2 days later. Experiments were replicated 2 to 5 times.

To detect cell surface markers, cells were extracted from methylcellulose and incubated with antibodies against Ly-6C/G (Gr-1) which were directly conjugated to PE and with biotin-conjugated antibodies against CD11b (Mac-1) which were detected with streptavidin conjugated to APC. All antibodies were obtained from Caltag. 0.2 µg of each antibody was used for staining 5×10^5^ cells in 50 µL of PBS for 30 mins at RT. Cells were washed twice, diluted in PBS, and analysed on a Cyan ADP MLE fluorescent analyser with Summit v4.3 software.

### Tumour formation assays

This study was carried out in strict accordance with the recommendations of the Home Office, and all efforts were made to minimize suffering. The protocol was approved by the local Ethics Committee at the University of Cambridge (License Number: 80/2000). Mice were obtained from Charles River Laboratories and maintained in individually ventilated cages. After infection of foetal liver cells, GFP expression was confirmed by flow cytometry. Infections ranged between 2–15% for Fbxo7 vectors and 4–20% for MSCV control vectors. Cells were washed with PBS prior to injection via the tail vein into irradiated (1000 rads) recipients. Tissues were harvested from euthanized mice, homogenized for FACS analysis to assess GFP expression, or fixed in 4% paraformaldehyde/PBS prior to embedding in paraffin wax. Tissue samples were sectioned and stained with hematoxylin and eosin (H&E), or with antibodies to CD3, [43] CD20 (Dako), or Fbxo7 as previously described [11].

### Cell cycle analysis

Analysis of the cell cycle distribution of cells was performed using Click-IT™ EdU Flow Cytometry Assay Kit (Invitrogen, A10202). After 9 days of growth in methylcellulose, cells were resuspended in 3 volumes of media (40% DMEM, 40% IMDM, 4 mM L-Glutamine, 0.1 mM β-mercaptoethanol, 16% fetal bovine serum, 4% WEHI-3B conditioned media, SCF 50 ng/mL, IL-3 10 ng/mL, IL-6 10 ng/mL). EdU (5-ethynyl-2′-deoxyuridine) was added to 15 µM, and cells were incubated at 37°C for 2 hrs prior to fixation in 2% paraformaldehyde. EdU incorporation was detected as per manufacturer's protocol, and cells were analysed on a Cyan ADP MLE fluorescent analyser (Dako).

### PCR analysis

PCR reactions for the p53 null transgene or the GFP gene present in the retrovirus were performed on DNA extracted from paraffin embedded tissues from control and Fbxo7 mice. PCR reactions were performed as follows: for amplification of the p53 transgene, reactions were 35 cycles of 92°C for 30 sec, 63°C for 30 sec, 72°C for 30 sec, using the forward primer 5′-AGCCTGAAGAACGAGATCAG-3′, and reverse primer 5′-TATACTCAGAGCCGGCCT-3′. For amplification of GFP sequences, a first-round PCR reaction was performed for 35 cycles of 92°C for 30 sec, 66°C for 30 sec, 72°C for 30 sec. The PCR product was purified using QIAgene PCR purification spin columns and used in a second-round PCR reaction of 30 cycles. For both reactions, the forward primer 5′-AGCCGCTACCCCGACCACAT, and the reverse primer 5′-CGGTTCACCAGGGTGTCGCC-3′ were used.

### Statistical analysis

All the *P* values were calculated by Student's t-test for two-tailed distribution and equal variance between samples using Microsoft Excel. Error is represented as the standard deviation. Kaplan-Meyer survival curves were calculated using MedCalc software. A logrank test was performed on the two survival curves, with a chi-square statistical test.
